# An Unusual Presentation of Peripartum Cardiomyopathy Complicated by a Pulmonary Embolism: A Case Report

**DOI:** 10.7759/cureus.51768

**Published:** 2024-01-06

**Authors:** Jawaher A Bubshait, Shanei A Shanei

**Affiliations:** 1 Internal Medicine, Bahrain Defense Force Hospital, Riffa, BHR; 2 Cardiology, Royal Medical Services, Bahrain Defense Force Hospital, Riffa, BHR

**Keywords:** pregnancy complications, cardiomyopathy, heart failure, pulmonary embolism, peripartum cardiomyopathy

## Abstract

Peripartum cardiomyopathy (PPCM) is a rare heart condition affecting women during pregnancy or up to five months postpartum whose incidence has been rising in recent years. It is characterized by a weakened left ventricle, which impairs the heart’s ability to pump blood effectively. Although the cause remains unknown, PPCM is a crucial consideration when evaluating heart failure in women of childbearing age.

In this report, we present the case of a young and healthy woman who, despite lacking any identifiable risk factors, developed symptoms of systolic heart failure while undergoing an abortion during her second pregnancy, which occurred within five months of delivering her first child. After thorough investigations, a diagnosis of PPCM was established. Her treatment took place at a tertiary hospital, with regular follow-up appointments scheduled. Unfortunately, due to non-compliance with her prescribed medications, she subsequently developed a pulmonary embolism, adding another layer of complexity to her situation.

This report showcases the critical role of early recognition and swift intervention in the patient’s return to stability.

## Introduction

Peripartum cardiomyopathy (PPCM) is a potentially life-threatening condition affecting pregnant women in their last month of pregnancy or up to five months after delivery. The timing of the occurrence of PPCM is still under discussion; Demakis et al. noted that most cases occur in the first weeks postpartum [1]. It is characterized by left ventricular dysfunction and systolic heart failure [2,3], despite the absence of pre-existing heart disease.

There are no documented statistics on the incidence of PPCM in the Kingdom of Bahrain and is generally rare, especially in the United States, Canada, and Europe. The published data for Arab countries remain limited, but a recent retrospective study conducted in Oman revealed an incidence of 1.02 per 1,000 deliveries during the eight-year study period [4].

Symptoms of PPCM vary and mostly represent the clinical manifestations of heart failure. Patients can present with dyspnea on exertion, orthopnea, and paroxysmal nocturnal dyspnea.

Findings on physical examination include tachycardia, respiratory distress, elevated jugular venous pressure, pulmonary rales, and peripheral edema. Bedside electrocardiography (ECG) typically reveals sinus tachycardia, and chest X-rays may indicate cardiomegaly and pulmonary venous congestion [2,3]. In some circumstances, PPCM is misdiagnosed, as the symptoms mimic the physiological changes of the third trimester of pregnancy, although the timing of onset differs [2].

Criteria for the diagnosis are assessed and a diagnosis is made when three criteria are present: (1) heart failure developing in the final month of pregnancy or within five months postpartum; (2) systolic dysfunction, characterized by reduced ejection fraction (EF) of less than 45%; (3) no other cause of heart failure with reduced EF being identified [1].

The pathophysiology behind the disease remains unknown, although many theories suggest the role of infectious myocarditis, autoimmunity, nutritional deficiencies, microchimerism, and hemodynamic stresses [2]. One theory associates PPCM with increased prolactin hormone release during the puerperal period, as the 16 kDa prolactin fragment was found to be overexpressed in women who developed PPCM, which could contribute to oxidative stress, inducing cell necrosis via several mechanisms [5].

Early recognition and treatment of PPCM are critical, as this potentially fatal condition can lead to life-threatening complications such as cardiopulmonary arrest, thromboembolic events, stroke (cerebrovascular accident), pulmonary edema, and even sudden cardiac death [6].

## Case presentation

A 20-year-old female patient, gravida 2, para 1, at 10 weeks 4 days of gestation visited the emergency room with a history of generalized fatigue in association with exertional shortness of breath, chest tightness, and palpitations. She had been experiencing symptoms for two months, which had started at the beginning of the current pregnancy and worsened over time. A few days before the presentation, the symptoms worsened and became more evident at rest and bedtime.

Her medical and surgical histories were unremarkable, with no history of smoking or alcohol consumption.

Her first pregnancy was spontaneous, and although she experienced mild hyperemesis gravidarum during her first trimester, she delivered vaginally without any complications. The patient was not using contraception. She became pregnant spontaneously three months after her previous delivery.

Her vital signs on arrival were blood pressure of 112/64 mmHg, heart rate of 120 bpm, respiratory rate of 19 breaths/min, temperature of 36.7 °C orally, and SpO2 99% on room air. A thorough examination, including respiratory, cardiac, and abdominal examination, uncovered no noteworthy features. ECG revealed sinus tachycardia. Laboratory investigations, including a complete blood count and measurement of urea and electrolyte levels, indicated normal levels. However, D-dimer was mildly elevated (2.59 ug/mL). Despite recommendations for further evaluation with serial cardiac enzymes and an echocardiogram, the patient chose to leave the hospital against medical advice.

After five days of the initial presentation, she revisited the emergency department with a one-day history of per-vaginal bleeding and the passage of a blood clot. Her vital signs were blood pressure of 106/86 mmHg, heart rate of 126 bpm, respiratory rate of 18 breaths/min, temperature of 36.5 °C orally, and SpO2 99% on room air. ECG indicated sinus tachycardia.

A vaginal examination revealed spotting only. A transvaginal scan (TVS) revealed a single intrauterine sac with a viable fetal pole with a crown rump length (CRL) of 8+5. After a few hours, the patient passed another blood clot, and TVS was repeated, revealing an empty uterus with an endometrial thickness of 2.1 cm. The patient was admitted to the gynecology ward, FIGO protocol was initiated, and the patient was treated accordingly.

Detailed laboratory investigations were carried out, the results of which are presented in Table 1. D-dimer and NT-proBNP levels were found to be elevated.

**Table 1:** Laboratory findings at time of presentation.

Laboratory test	Value	Unit	Normal range
White blood cells	7.2	x10^9^/L	4.00-11.00
Hemoglobin	11.4	g/dL	12.0-16.0
Platelet count	193.0	x10^9^/L	150-450
INR	1.23		0.98-1.13
PT	16.7	sec	13-15
APTT	34.9	sec	28-45
D-Dimer	4.24	ug/mL	0-0.5
Fibrinogen	2.79	g/L	2-4
NT-proBNP	1,813	pg/mL	<300
CK-MB	13.20	IU/L	0-25
Troponin-I	0.00	ug/L	0-0.3

**Figure 1:**
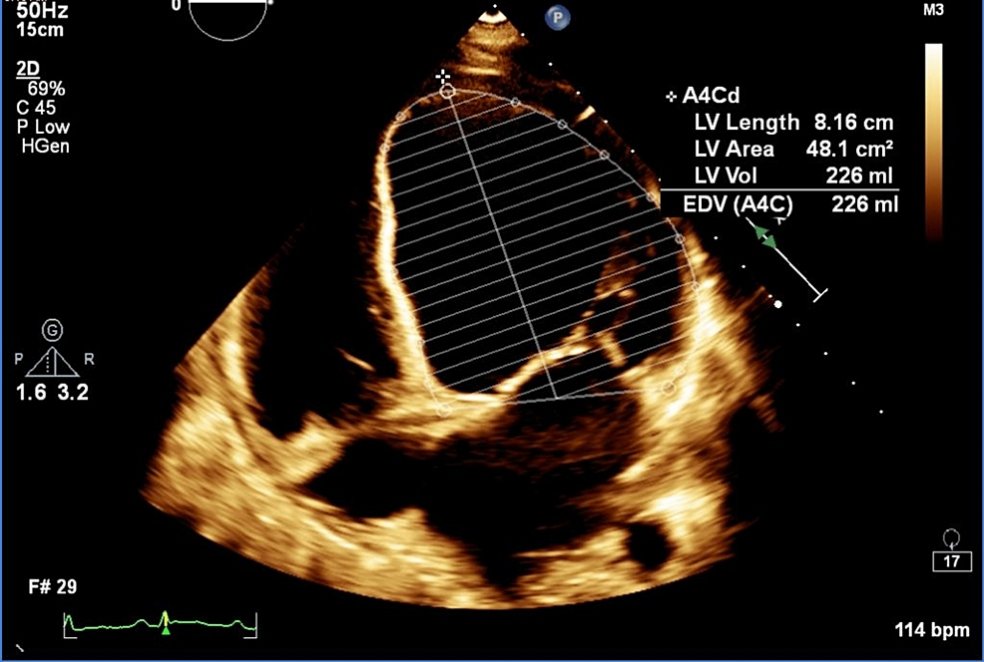
Echocardiogram images at initial presentation The ECG depicts severely dilated LA and LV, and severe global hypokinesia with severe LV dysfunction. The estimated ejection fraction is 25%. The ejection fraction by Simpon’s method is 23%. GLS using speckle tracking with EPIQ CVx machine (Phillips Healthcare, Best, Netherlands) is -6.3%. EPSS 34mm, MAPSE 10mm. Myocardial performance index is 1.07. LA: left atrium; LV: left ventricle; GLS: global longitudinal strain; EPSS: E-point septal separation; MAPSE: mitral annular plane systolic excursion

She remained tachycardic, and the cardiology team was consulted. An echocardiogram was ordered, the results of which were as follows: severely dilated left ventricle with severe global hypokinesia to akinesia; estimated ejection fraction (EF) of <25%, with EF of 24% obtained by Simpson’s method; indeterminate diastolic function; non-invasive cardiac index of 1.6 L/m2; tethering and annulus dilation of mitral valve with moderate to severe eccentric mitral regurgitation (Figure 1). CXR was not significant. Hence, the patient was diagnosed with decompensated symptomatic peripartum cardiomyopathy with low cardiac output.

The patient passed the remainder of the product of conception spontaneously and was strongly advised regarding avoidance of pregnancy.

The patient started on the standard anti-heart failure medications including diuretics, spironolactone, SGLT2i inhibitors, and beta blockers, as well as anticoagulants. In addition, Entresto was intended to be started but could not due to persistent hypotension. On the second day, the patient developed hypotension requiring inotropic support. 

Further investigations to find a reversible cause were carried out, including infectious and rheumatological tests, which all turned out to be negative.

Transfer to a tertiary cardiac center was arranged for life-saving support. Her medications were optimized during admission, resulting in a gradual improvement of her symptoms. An echocardiogram was performed before discharge, indicating an LVEF of 20%, diffuse hypokinesia, a moderately dilated left atrium, mild to moderate mitral regurgitation, mild tricuspid, and pulmonary regurgitation, SPAP of 26.6 mmHg, and trivial pericardial effusion globally. A strain echo indicated global impaired longitudinal function (GLS -8). A cardiac MRI was performed and revealed a right ventricular thrombus, the patient was started on the anticoagulant edoxaban 60 mg.

She was discharged from the tertiary center with multiple anti-heart failure medications including spironolactone, furosemide, empagliflozin, ivabradine hydrochloride, and bisoprolol, in addition to anti-coagulant, with a plan to follow her as an outpatient.

After a couple of weeks, she presented to the tertiary center emergency clinic with a history of persistent shortness of breath and palpitations, which started after she had stopped all of her medications three days earlier, and the D-dimer was elevated. CT pulmonary angiography was performed, which revealed acute sub-segmental pulmonary embolism (PE). Echocardiography was repeated, revealing LVEF of 15-20%, diffuse LV hypokinesis, and normal RV function, with the presence of thrombi measuring 1.2 cm×12 cm and 1 cm×1.3 cm in the basal anterior wall and apex, respectively. She was admitted to the wards and her anticoagulant was switched to rivaroxaban 15 mg BD initially. Following a marked improvement, the patient was discharged home with a strict medication regimen of anticoagulants and anti-heart failure medications. She was reminded of the importance of close follow-up visits for optimal monitoring.

During her follow-up appointment three weeks post-discharge, the patient remained asymptomatic, with no signs of heart failure or PE progression. Her blood pressure was 103/79 mmHg and her heart rate was 115 bpm. The cardiovascular examination was unremarkable. Her dose of bisoprolol was increased to 2.5 mg once daily. Furthermore, the dose of rivaroxaban was changed to 20 mg once daily on the basis of the PE protocol.

Two months later, the patient was referred for pre-operative clearance for laparoscopic cholecystectomy, as she experienced recurrent episodes of acute cholecystitis. Echocardiography was repeated, indicating static LVEF of 15%, no RV clot, and moderate MR and TR appreciated. Benefits and risks were measured, and the patient decided to undergo surgery. During the pre-operative period, rivaroxaban was withheld and she experienced worsening shortness of breath; thus, she was taken to the ICU, where a repeated CTPA turned out to be negative. High-risk consent was taken, and surgery went uneventfully. Her post-operative hospital stay was uneventful, and she was discharged in a stable condition.

The patient was scheduled for regular follow-ups in the cardiology outpatient department. Her first follow-up was uneventful, cardiovascular examination was normal, and repeated echocardiography revealed static findings. The patient was advised to comply with medications and further follow-ups.

A couple of days later, she had repeated visits to the emergency room, complaining of chest pain and persistent shortness of breath. After a proper evaluation, the diagnosis of viral pneumonia was made and the patient was opted for admission given her high-risk status. Echocardiography was repeated and showed severe global hypokinesia of LV with severe LV dysfunction, estimated LVEF <15%. RV of normal size with reduced systolic function and two large RV thrombi seen measuring 26x14 mm and 14x9 mm. In addition, moderate eccentric mitral regurgitation and severe tricuspid regurgitation with mild pulmonary hypertension (RVSP 40mmHg). The inferior vena cava was dilated with no respiratory collapsibility. CTPA was performed, which ruled out PE. Further deterioration of the patient's clinical condition led to her being transferred to the ICU, intubated, and kept on maximum inotropic support. Unfortunately, the patient became pulse-less, resuscitation attempts failed and death was declared. 

## Discussion

Although PPCM is relatively uncommon, its incidence is rising. This may be related to advanced maternal age, increased rates of multifetal pregnancies, and possibly increased recognition and awareness of the disease [2].

The incidence has been found to be highest among women of African ethnicity: a study conducted in 2007 revealed the incidence among African American women to be 2.9-fold and 7-fold those of White and Hispanic women, respectively [7].

About 1,000 to 1,400 women develop the condition in the United States each year. PPCM is more common in some countries than others and may be related to differences in diet, lifestyle, other medical conditions, or genetics. Its incidence has been reported to be as high as 1 in 100 deliveries in Nigeria, 1 in 300 deliveries in Haiti, and as low as 1 in 20,000 deliveries in Japan [6]. A retrospective study from Oman revealed an incidence of 1.02 per 1,000 deliveries over eight years [4].

The occurrence of PPCM has been linked to several risk factors. Advanced maternal age has been strongly associated with PPCM; according to Gunderson et al., one of the strongest independent predictors was maternal age beyond 40 years [8]. Therefore, the patient’s young age of 21 at pregnancy onset is noteworthy, suggesting that age may not be the sole factor and that further investigation into other potential contributing factors is advisable.

In addition to advanced maternal age, pre-existing hypertension, multiple gestation, and pregnancy-related hypertensive disorders have been found to be associated with an increased risk of PPCM [8].

Previous studies on the diagnosis of PPCM patients have reported abnormal electrocardiograms indicating left ventricular hypertrophy and ST-T changes [9]; chest X-rays revealing cardiomegaly and often pulmonary congestion [3]; and echocardiograms indicating LVEF <45% [6]; as well as ventricular dilatation, ventricular dysfunction, valvular dysfunction, atrial dilatation, and pulmonary hypertension [2].

In the present case, despite initial findings revealing no concerning signs in the clinical exam or basic lab investigations (except mild elevated D-dimer), the patient’s case unfolded rapidly over five days. ECG revealed sinus tachycardia, and TVS identified blood clots. Subsequent echocardiography clearly indicated decompensated symptomatic peripartum cardiomyopathy, characterized by a severely dilated left ventricle with impaired function, valve leakage, and, ultimately, low cardiac output.

PPCM treatment prioritizes symptom control with safe, pregnancy-compatible medications. Diuretics and nitrates manage fluid buildup, while ACE inhibitors/ARBs (postpartum only) and beta blockers address hormonal imbalances. Anticoagulants (low-molecular-weight heparin) prevent blood clots, avoiding teratogenic warfarin during pregnancy [2-9].

Two randomized control trials have been performed to assess the effect of bromocriptine and levosimendan. Bromocriptine, a prolactin inhibitor, showed promise with a 31% LVEF improvement and better survival, whereas levosimendan, a heart support drug, provided no clear benefits. A non-randomized study suggested that pentoxifylline, a TNF-alpha inhibitor, may reduce PPCM complications [7].

A German study found that ivabradine, a drug that lowers the heart rate, improved outcomes in PPCM patients with high resting heart rates and low blood pressure, suggesting that it may be an alternative to beta blockers in such cases [9].

In severe and complicated cases, mechanical circulatory support and a heart transplant may be needed. Intra-aortic balloon pumps and LV assist devices have been approved for use as bridges for recovery in fulminant cases and for heart transplantation [10].

Demakis et al. have reported the recurrence risk and cause of death in PPCM patients. They found that early heart size recovery in 14 patients predicted good outcomes, with only two having PPCM recurrence in later pregnancies. In contrast, persistent cardiomegaly in 13 patients indicated a poorer prognosis, with three dying from repeated heart failure [3]. Another study conducted in California identified PPCM as the cause of death for 23% of pregnancy-related fatalities, highlighting its significant impact on maternal mortality [2]. A Nigerian study found high rates of ventricular thrombi (21.4% left, 16.7% right) in PPCM patients, likely due to hypercoagulability during pregnancy, although an Omani study reported a much lower (3.1%) thromboembolic event rate [4].

Although formal recommendations are lacking for future pregnancy after PPCM, the potential for lasting heart damage grows with each pregnancy. Full recovery before attempting to become pregnant again is crucial, and any subsequent pregnancy requires high-risk classification and close monitoring [10].

Although our patient lacked typical risk factors such as race, age at presentation, and multiple pregnancies, and her symptoms began early in her second pregnancy, we suspect a connection to her recent first pregnancy due to the timely proximity within the postpartum period.

## Conclusions

Peripartum cardiomyopathy’s chilling grip claims too many lives, as evidenced by our young patient’s tragic trajectory. Although multidisciplinary management in specialized centers offers a lifeline, gaps in understanding and treatment remain. Additional challenge to address is the patient’s understanding and compliance with medications and follow-up visits. Moving forward, it is necessary to understand in greater depth the burden of PPCM in communities, thus empowering early intervention and improving lives of mothers. 
